# Supplementary material for: In Silico Food-Drug Interaction: A Case Study of Eluxadoline and Fatty Meal
Source: Int J Mol Sci. 2020 Nov 30;21(23):9127. doi: 10.3390/ijms21239127 (PMC7731208; doi:10.3390/ijms21239127)
Supplement: Supplementary file 1 [file ijms-21-09127-s001.pdf]

Supplementary Material

In Silico Food-Drug Interaction: the Case Study of Eluxadoline and Fatty Meal

Annalisa Maruca [1,2#], Antonio Lupia [2#], Roberta Rocca [2,3*], Keszthelyi Daniel [4], Maura Corsetti [5,6] and Stefano Alcaro [1,2]

1 Dipartimento di Scienze della Salute, Università "Magna Græcia" di Catanzaro, Campus "S. Venuta", Viale Europa, 88100, Catanzaro, Italy; maruca@unicz.it, alcaro@unicz.it

2 Net4Science Academic Spin-Off, Università "Magna Græcia" di Catanzaro, Campus "S. Venuta", Viale Europa, 88100, Catanzaro, Italy; antonio.lupia@net4science.com;

3 Dipartimento di Medicina Sperimentale e Clinica, Università "Magna Græcia" di Catanzaro, Campus "S. Venuta", Viale Europa, 88100, Catanzaro, Italy; rocca@unicz.it

4 Division Gastroenterology-Hepatology, Department of Internal Medicine, NUTRIM School for Nutrition and Translational Research in Metabolism, Maastricht University Medical Center, Universiteitssingel 50, Maastricht, NL; daniel.keszthelyi@maastrichtuniversity.nl

5 NIHR Nottingham Biomedical Research Centre, Nottingham University Hospitals NHS Trust, UK; Maura.Corsetti@nottingham.ac.uk

6 University of Nottingham and Nottingham Digestive Diseases Centre, School of Medicine, University of Nottingham, Nottingham, UK.

* Correspondence: rocca@unicz.it

† These authors contributed equally.

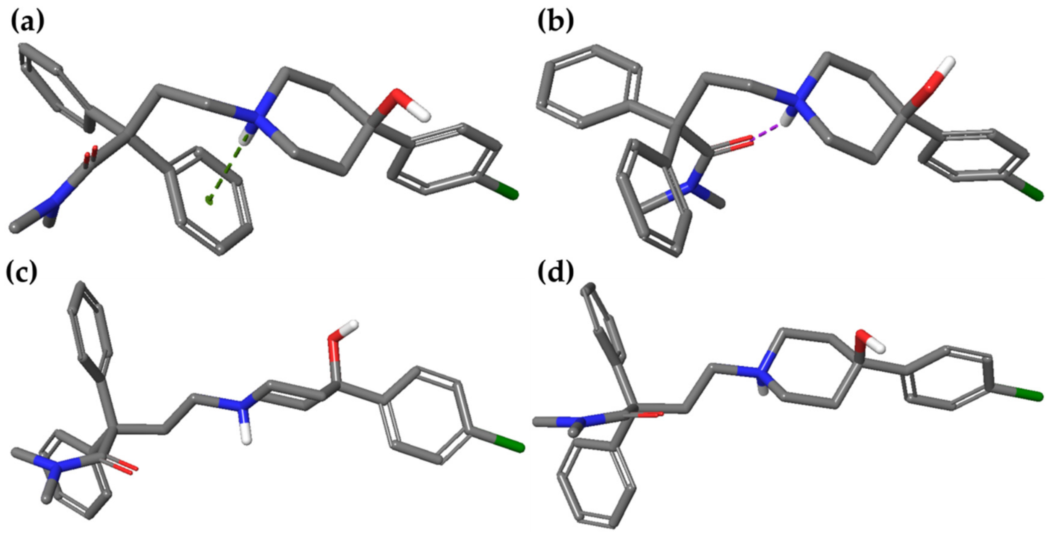

Figure S1. Three-dimensional structures of the four best energy minima (%B> 10%) of loperamide (L), obtained after MC simulation in water. Intramolecular interactions are shown as green (π-cation) and purple (H-bond) dash lines. ***(a)*** **L-MC1w**, ***(b)*** **L-MC2w**, ***(c)*** **L-MC3w** and **(d) L-MC4w**.

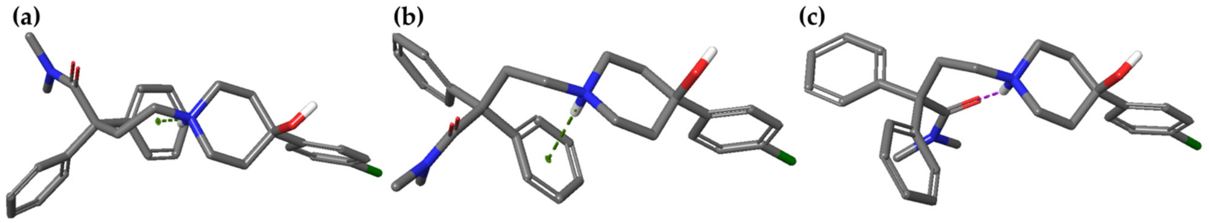

Figure S2. Three-dimensional structures of the three best energy minima (%B> 10%) of eluxadoline (E), obtained after MC simulation in octanol. Intramolecular interactions are shown as green (π-cation) and purple (H-bond) dash lines. ***(a)*** **L-MC1o**, ***(b)*** **L-MC2o** and ***(c)*** **L-MC3o**.

Table S1. RMSd values obtained from the comparison of the most probable energy minima for loperamide obtained after MC simulations both solvents. RMSd values are reported in Å, and they are calculated on the heavy atoms of loperamide.

**RMSd**	**L-MC1o**	**L-MC2o**	**L-MC3o**
**L-MC1w**	2.49	0.11	2.55
**L-MC2w**	2.72	2.57	0.19
**L-MC3w**	3.05	2.62	1.89
**L-MC4w**	2.28	2.68	2.15

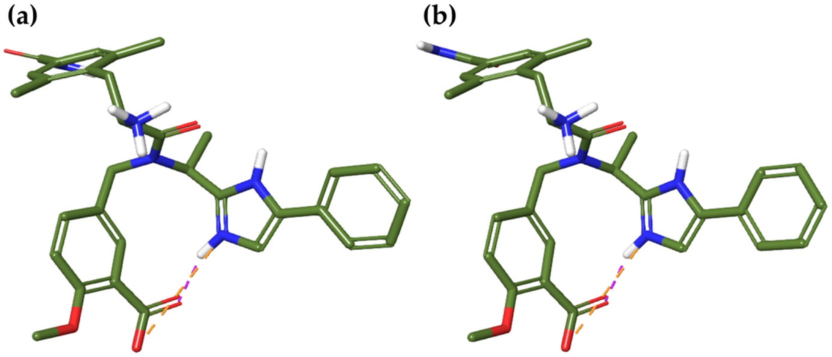

Figure S3. Three-dimensional structures of the best energy minima (%B> 10%) of eluxadoline, obtained after MC simulation in water at pH 6 (E1). Intramolecular interactions are shown orange (salt bridges), and purple (H-bond) dash lines. ***(a)*** **E1-MC1w** and ***(b)*** **E1-MC2w**.

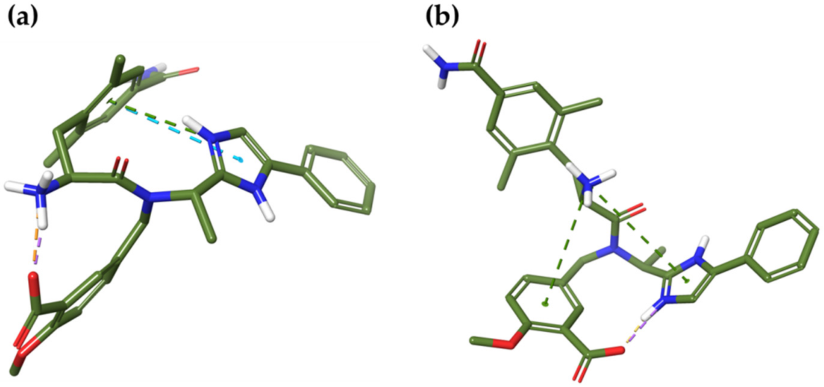

Figure S4. Three-dimensional structures of the best energy minima (%B> 10%) of eluxadoline, obtained after MC simulation in octanol at pH 6 (E1). Intramolecular interactions are shown orange (salt bridges), purple (H-bond), cyan (π-π) and green (π-cation) dash lines. ***(a)*** **E1-MC1o** and ***(b)*** **E1-MC2o**.

Table S2. RMSd values obtained from the comparison of the most probable energy minima for E1 obtained after MC simulations both solvents. RMSd values are reported in Å, and they are calculated on the heavy atoms of E1.

**RMSd**	**E1-MC1o**	**E1-MC2o**
**E1-MC1w**	3.46	2.16
**E1-MC2w**	3.46	2.15

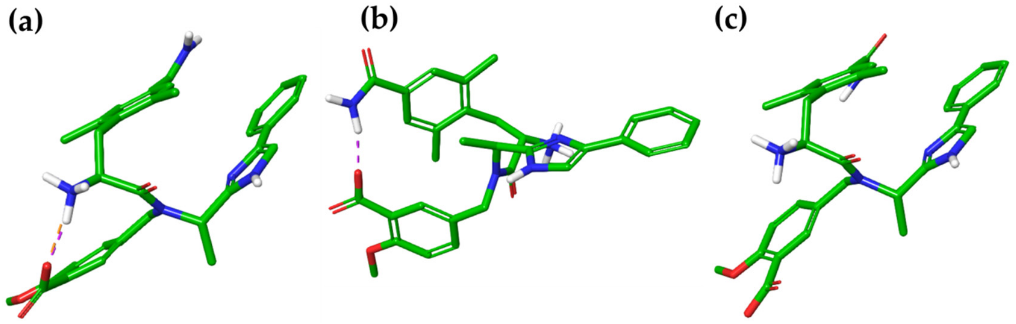

Figure S5. Three-dimensional structures of the best energy minima (%B> 10%) of eluxadoline, obtained after MC simulation in water at pH 7 (E2). Intramolecular interactions are shown orange (salt bridges) and purple (H-bond) dash lines. ***(a)*** **E2-MC1w**, ***(b)*** **E2-MC2w** and ***(c)*** **E2-MC3w**.

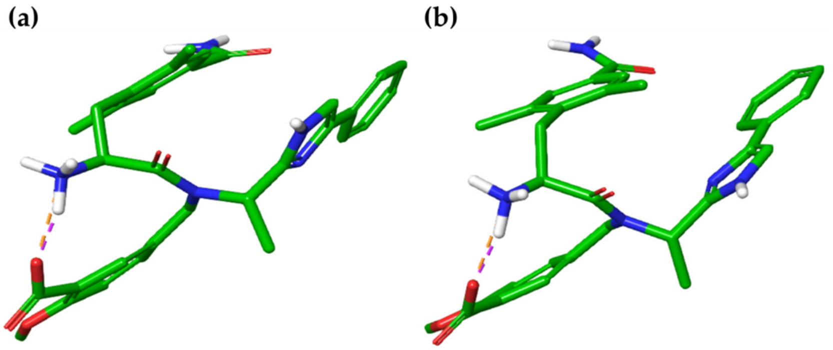

Figure S6. Three-dimensional structures of the best energy minima (%B> 10%) of eluxadoline, obtained after MC simulation in octanol at pH 7 (E2). Intramolecular interactions are shown orange (salt bridges) and purple (H-bond) dash lines. ***(a)*** **E2-MC1o** and ***(b)*** **E2-MC2o**.

Table S3. RMSd values obtained from the comparison of the most probable energy minima for E2 obtained after MC simulations both solvents. RMSd values are reported in Å, and they are calculated on the heavy atoms of E2.

**RMSd**	**E2-MC1o**	**E2-MC2o**
**E2-MC1w**	0.56	0.49
**E2-MC2w**	3.49	3.44
**E2-MC3w**	0.69	0.60

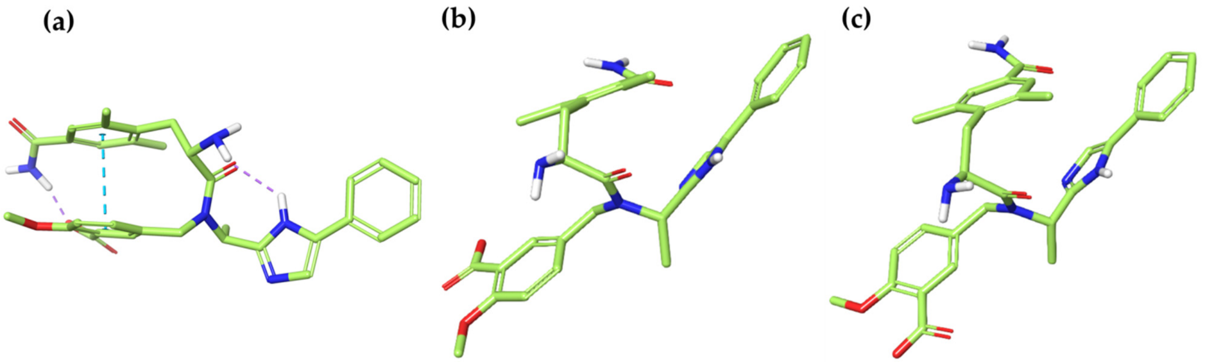

Figure S7. Three-dimensional structures of the best energy minima (%B> 10%) of eluxadoline, obtained after MC simulation in water at pH 8 (E3). Intramolecular interactions are shown orange (salt bridges), purple (H-bond), cyan (π-π) and green (π-cation) dash lines. ***(a)*** **E3-MC1w**, ***(b)*** **E3-MC2w** and ***(c)*** **E3-MC3w**.

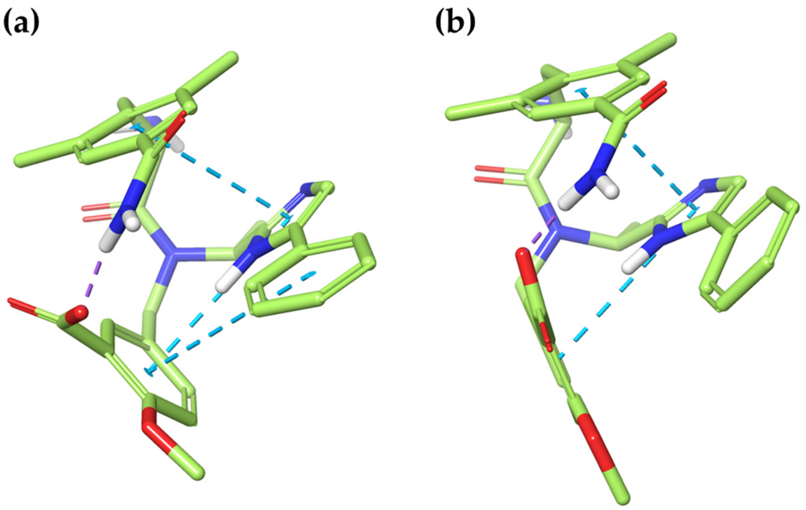

Figure S8. Three-dimensional structures of the best energy minima (%B> 10%) of eluxadoline, obtained after MC simulation in octanol at pH 8 (E3). Intramolecular interactions are shown in purple (H-bond) and cyan (π-π) dash lines. ***(a)*** **E3-MC1o** and ***(b)*** **E3-MC2o**.

Table S4. RMSd values obtained from the comparison of the most probable energy minima for E3 obtained after MC simulations both solvents. RMSd values are reported in Å, and they are calculated on the heavy atoms of E3.

RMSd	E3-MC1o	E3-MC2o
**E3-MC1w**	4.61	4.80
**E3-MC2w**	4.35	4.45
**E3-MC3w**	4.45	4.40

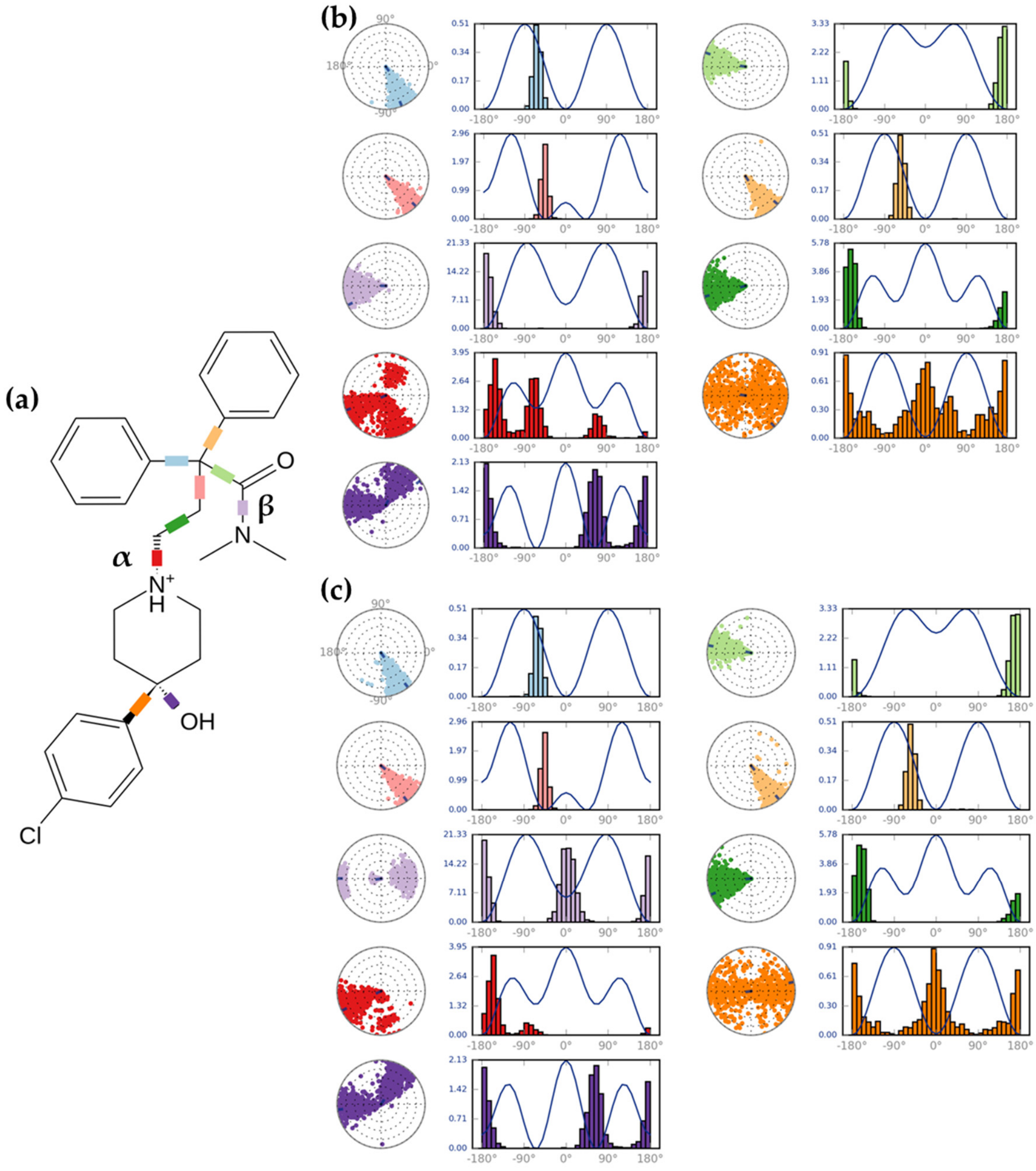

Figure S9. (a) Two-dimensional representation of loperamide, showing the rotatable bonds (RBs) encoded by different colors. **(b-c)** Dial (or radial) and bar plots of the ligand torsions during the MD simulation in water and octanol, respectively. The dial plot describes the conformation of the torsion throughout the simulation. The beginning of the simulation is in the centre of the radial plot and the time evolution is plotted radially outwards. The bar plots summarize the data on the dial plots, by showing the probability density of the torsion.

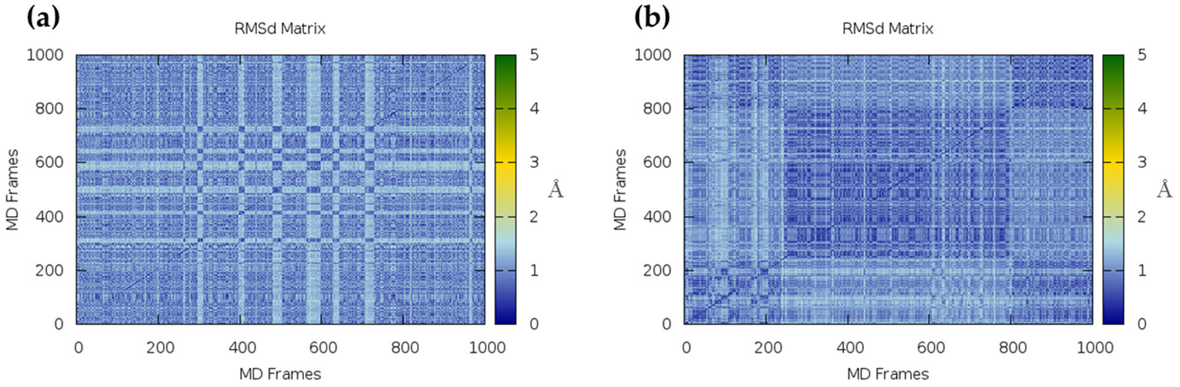

Figure S10. RMSd matrices calculated on loperamide heavy atoms among all the saved structures throughout the whole MDs in ***(a)*** water and ***(b)*** octanol, respectively.

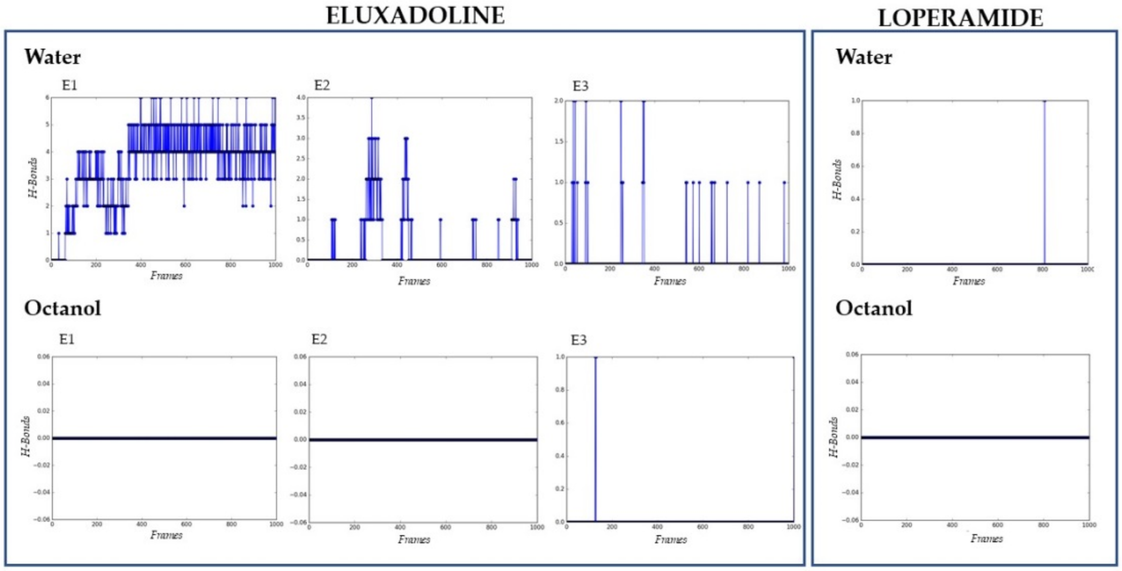

Figure S11. The H-bond plot representing the total number of H-bonds formed between two molecules of eluxadoline or loperamide in water or octanol. For eluxadoline, all the three most important protonation states (E1, E2 and E3) were considered and submitted to different MD simulations.

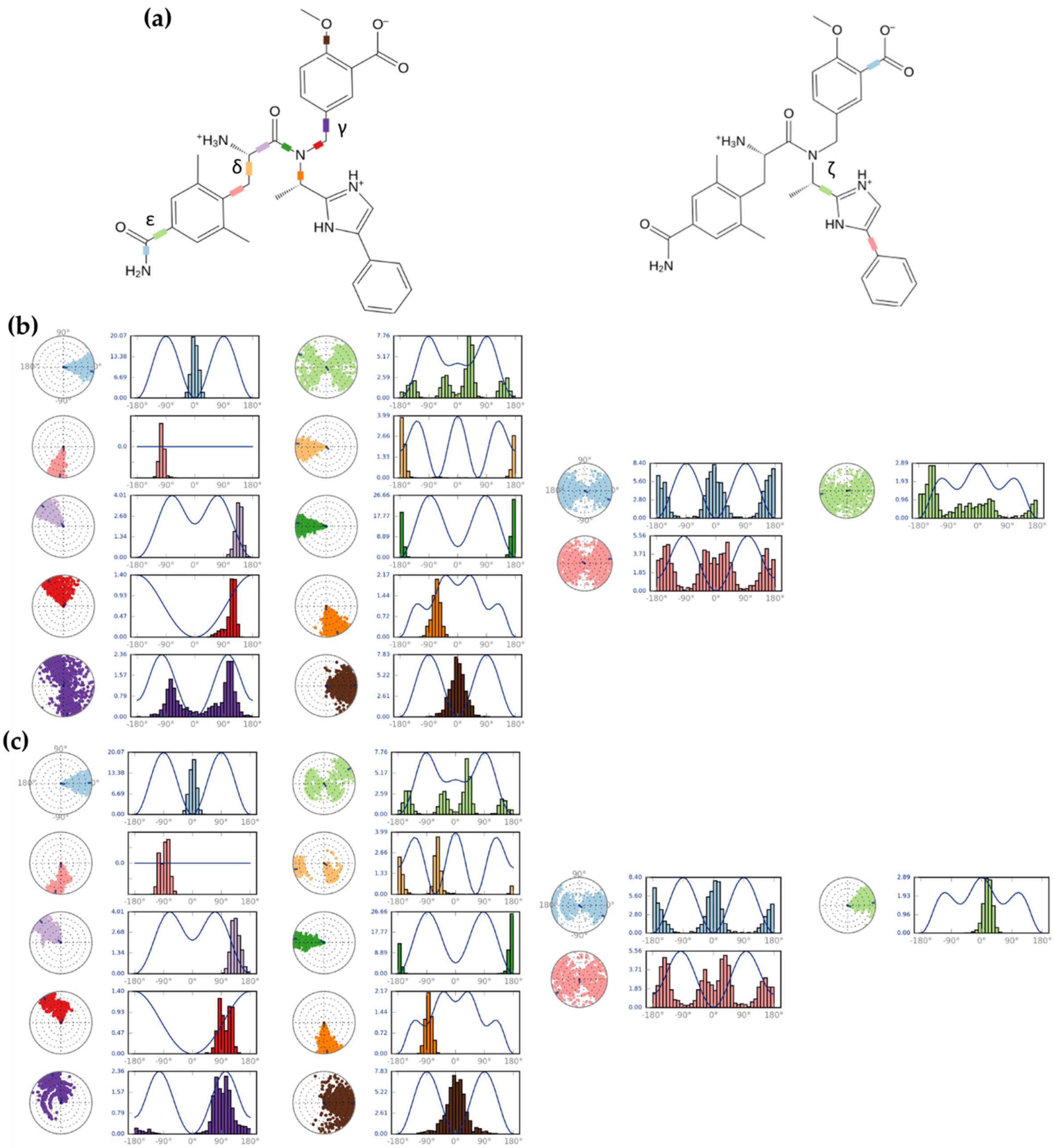

Figure S12. (a) Two-dimensional representation of E1, showing the rotatable bonds encoded by different colors. **(b-c)** Dial (or radial) and bar plots of the ligand torsions during the MD simulation in water and octanol, respectively. The dial plot describes the conformation of the torsion throughout the course of the simulation. The beginning of the simulation is in the centre of the radial plot and the time evolution is plotted radially outwards. The bar plots summarize the data on the dial plots, by showing the probability density of the torsion**.**

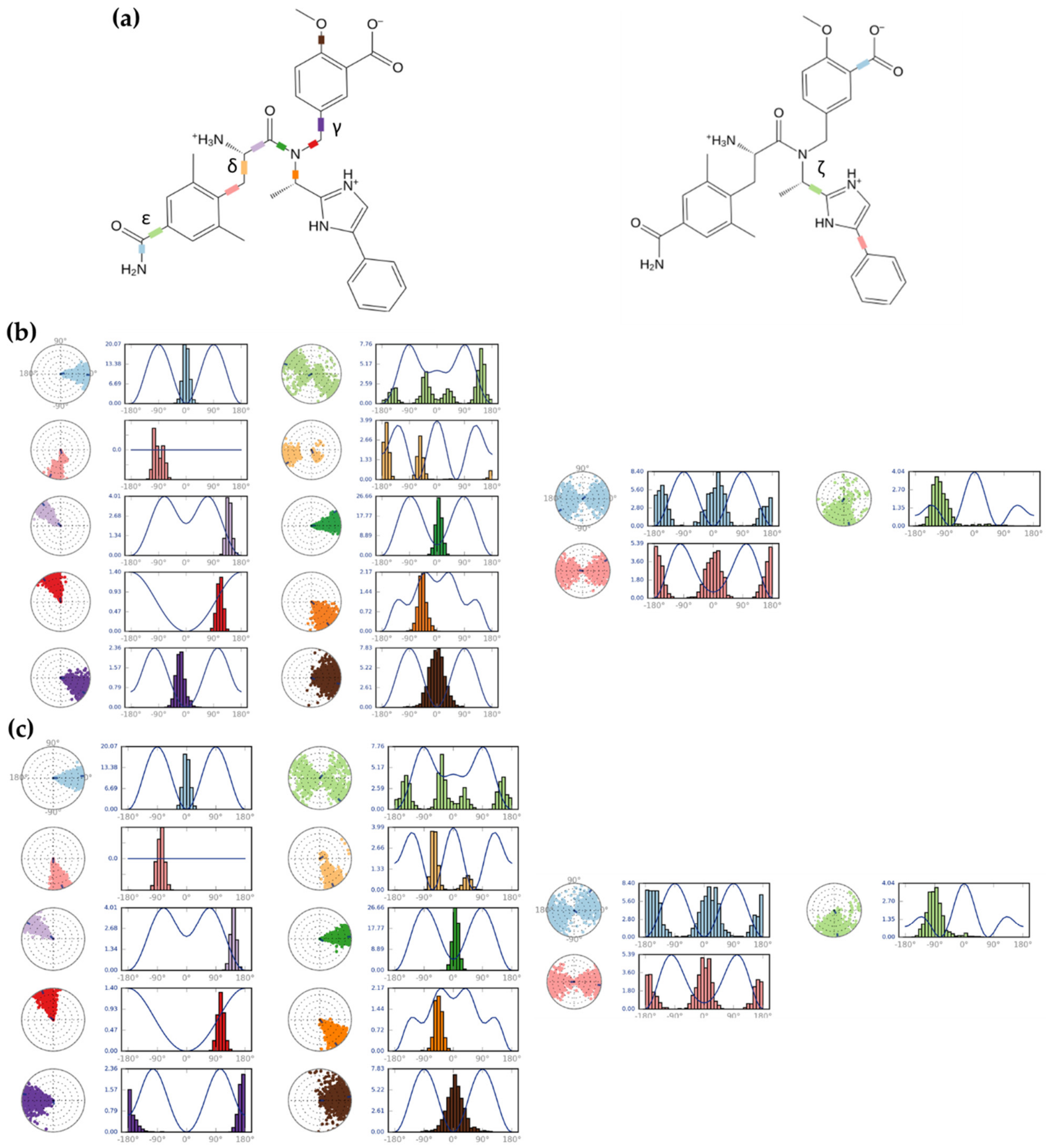

Figure S13. (a) Two-dimensional representation of E2, showing the rotatable bonds encoded by different colors. **(b-c)** Dial (or radial) and bar plots of the ligand torsions during the MD simulation in water and octanol, respectively. The dial plot describes the conformation of the torsion throughout the course of the simulation. The beginning of the simulation is in the centre of the radial plot and the time evolution is plotted radially outwards. The bar plots summarize the data on the dial plots, by showing the probability density of the torsion**.**

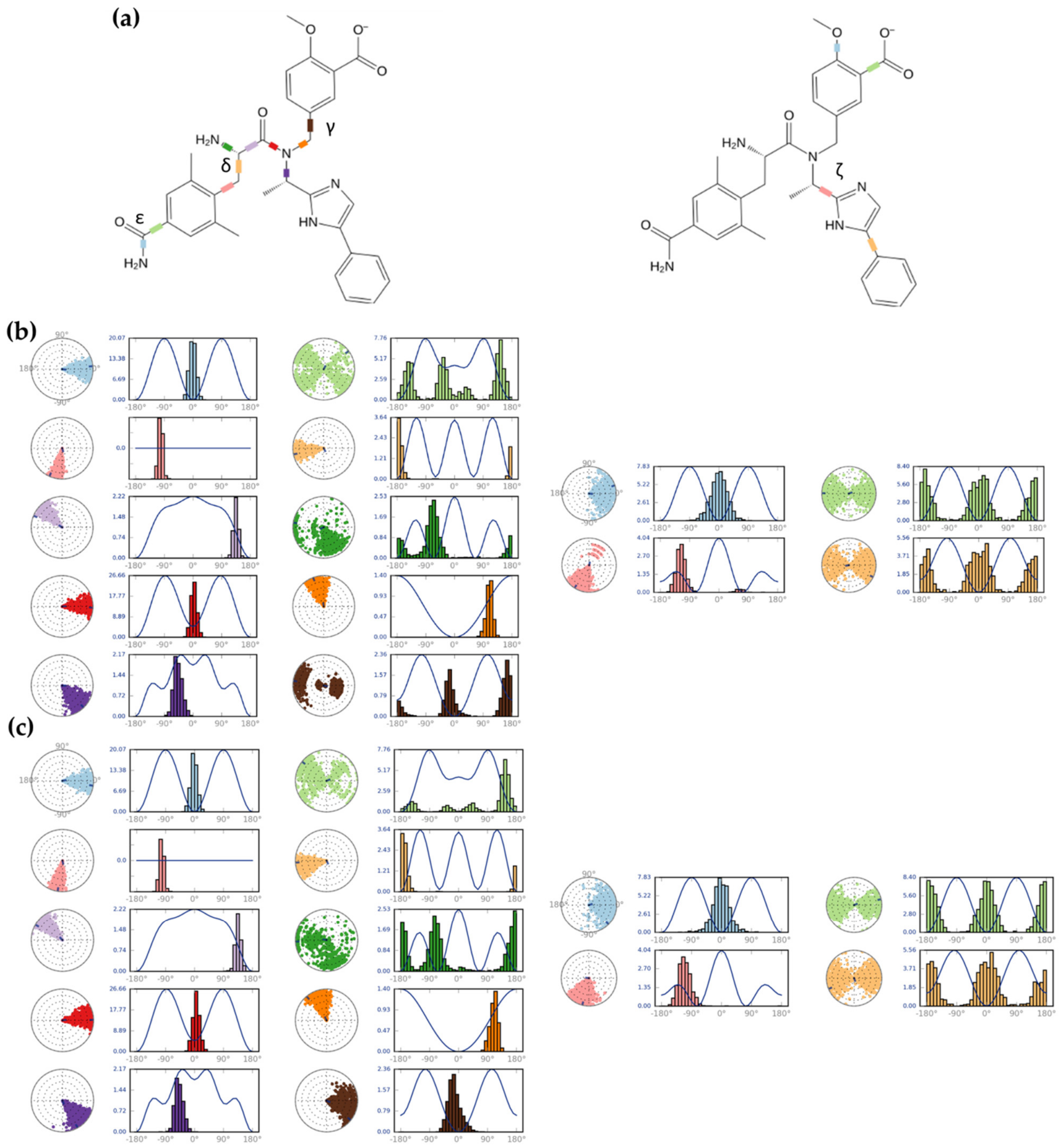

Figure S14. ***(a)*** Two-dimensional representation of E3, showing the rotatable bonds encoded by different colors. ***(b-c)*** Dial (or radial) and bar plots of the ligand torsions during the MD simulation in water and octanol, respectively. The dial plot describes the conformation of the torsion throughout the simulation. The beginning of the simulation is in the centre of the radial plot and the time evolution is plotted radially outwards. The bar plots summarize the data on the dial plots, by showing the probability density of the torsion.

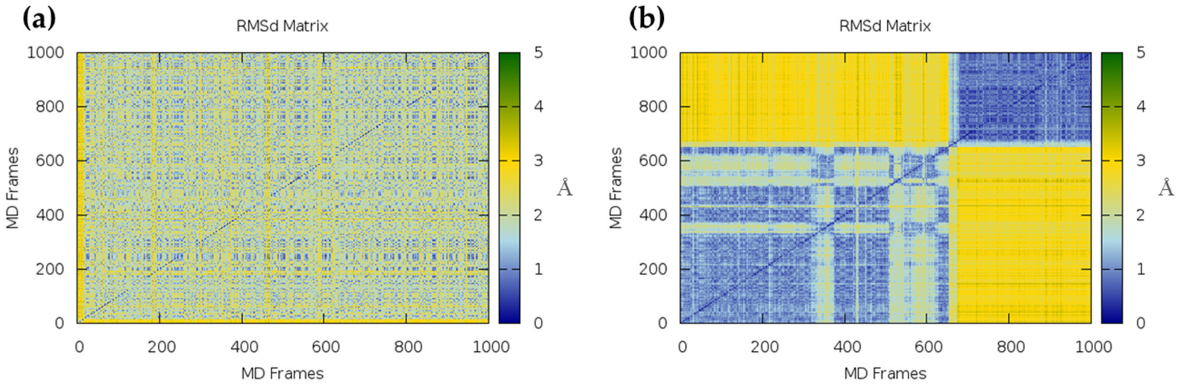

Figure S15. RMSd matrices calculated on E1 heavy atoms among all the saved structures throughout the whole MDs in *(a)* water and *(b)* octanol, respectively.

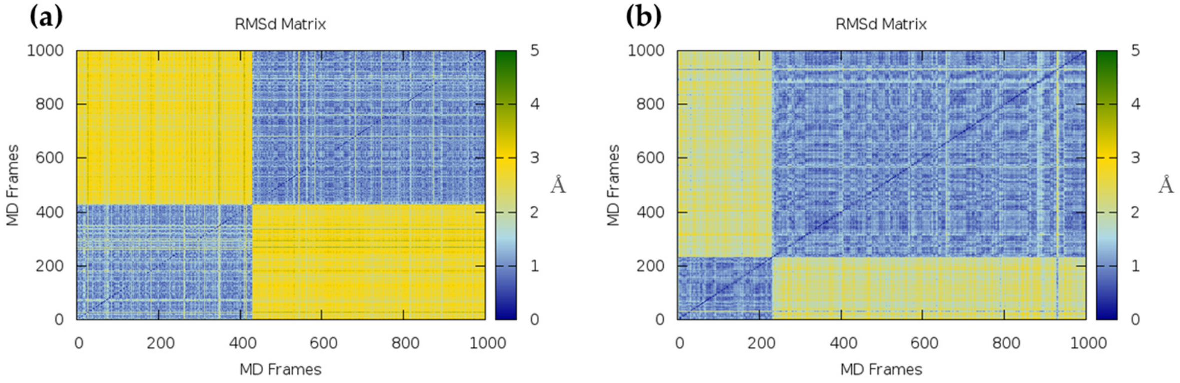

Figure S16. RMSd matrices calculated on E3 heavy atoms among all the saved structures throughout the whole MDs in *(a)* water and *(b)* octanol, respectively.

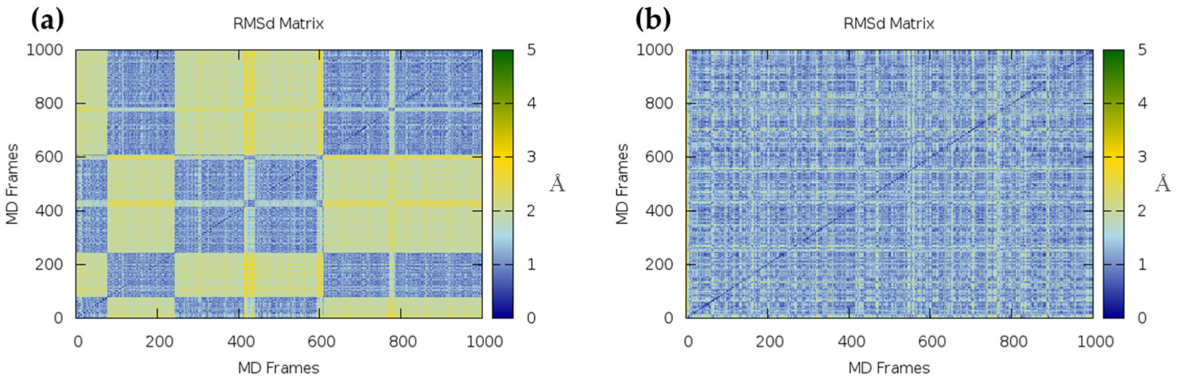

Figure S17. RMSd matrices calculated on E2 heavy atoms among all the saved structures throughout the whole MDs in *(a)* water and *(b)* octanol, respectively.
